# Delayed-onset endophthalmitis associated with corneal suture infections

**DOI:** 10.1186/1869-5760-3-51

**Published:** 2013-06-11

**Authors:** Christopher R Henry, Harry W Flynn Jr, Darlene Miller, Amy C Schefler, Richard K Forster, Eduardo C Alfonso

**Affiliations:** 1 Department of Ophthalmology, Bascom Palmer Eye Institute, University of Miami Miller School of Medicine, 900 NW 17th Street, Miami, FL 33136, USA; 2 Retina Consultants of Houston, Houston, TX 77030, USA

**Keywords:** Endophthalmitis, Corneal sutures, Suture abscess, Infectious keratitis, *Streptococcus*

## Abstract

**Background:**

The purpose of the current study was to report the microbiology, risk factors, and treatment outcomes in patients with delayed-onset endophthalmitis associated with corneal suture infections. For this retrospective consecutive case series, a search of the ocular microbiology department database was performed to identify all patients with positive corneal and intraocular cultures (anterior chamber and/or vitreous) between 01 January 1995 and 01 January 2010. A subset of patients with a history of corneal suture infections and delayed-onset endophthalmitis was identified.

**Results:**

Over the 15-year period of the study, 68 patients were identified to have both positive corneal and intraocular cultures. Among them, six patients were identified to have a culture-proven, delayed-onset endophthalmitis that developed from a culture-positive corneal suture infection. All of the patients in the current study were using topical corticosteroids at the time of diagnosis. In four of six patients, there was documented manipulation of a suture before the development of endophthalmitis. *Streptoccocus* was identified as the causative organism in five of six patients in the current study. All of the *Streptoccocus* isolates were sensitive to vancomycin. The single case of *Serratia marcescens* endophthalmitis was sensitive to amikacin, ceftazidime, ciprofloxacin, gentamicin, and tobramycin. Treatment modalities varied and were guided by the attending ophthalmologist depending upon clinical presentation. One patient with severe *Streptococcus pyogenes* keratitis and endophthalmitis underwent a primary enucleation after developing a wound dehiscence. Of the remaining five patients, all received topical and intravitreal antibiotics. Therapeutic penetrating keratoplasty was performed in three patients. Pars plana vitrectomy was performed in two patients. Visual acuity outcomes ranged from 20/150 to no light perception.

**Conclusions:**

In the current study, *Streptococcus* was isolated in nearly all patients with delayed-onset endophthalmitis associated with corneal suture infections. Topical steroid use and suture manipulation were identified as associated factors for developing endophthalmitis. Visual acuity outcomes were poor despite the prompt recognition of endophthalmitis and appropriate antibiotic therapy.

## Background

Corneal suture-related complications, particularly following penetrating keratoplasty, are well-known. Reported hazards of corneal sutures include suture erosions, sterile corneal infiltrates, and infectious keratitis, among others [1-6]. Additionally, there are risks associated with corneal suture removal including wound leakage, wound dehiscence, astigmatic changes, and even graft rejection [1-6]. Despite this, relatively few cases of corneal suture-related endophthalmitis have been reported in the literature [7-12].

The purpose of the current study is to describe a consecutive series of patients with delayed-onset endophthalmitis associated with corneal suture infections and to report the associated microbiology, risk factors, and treatment outcomes in these patients.

## Methods

Institutional Review Board approval was obtained from the University of Miami Miller School of Medicine Sciences Subcommittee for the Protection of Human Subjects. For this retrospective consecutive case series, a search of the ocular microbiology department database was performed to identify all patients with positive corneal and intraocular cultures between 01 January 1995 and 01 January 2010. Medical records were further reviewed to identify a subset of patients with a history of corneal suture infection that later developed endophthalmitis. To be included in the current study, patients were required to have positive corneal cultures from a suture infection or suture abscess and positive intraocular cultures from fluid from the anterior chamber and/or vitreous. The same organism was required to be positive from both corneal and intraocular cultures. Endophthalmitis was defined by the presence of severe intraocular inflammation as well as positive intraocular cultures. The current study represents a subgroup analysis of a previously reported consecutive series on keratitis-associated endophthalmitis [8].

All corneal cultures in the current study were obtained via corneal scraping along the involved suture site and were plated directly onto several different culture media, including chocolate agar, 5% sheep blood agar, and Sabouraud agar. All anterior chamber cultures in the current study were obtained at the time of penetrating keratoplasty. Vitreous cultures were obtained either at the time of vitreous tap and inject or during pars plana vitrectomy. Sutures were sent for cultures in selected cases. Specimens underwent incubation as described previously by our group [8]. All cultures were read and classified by Ocular Microbiology Department staff. Antibiotic sensitivities were performed on all gram-positive and gram-negative bacteria.

After analyzing the microbiology records, the corresponding medical records were reviewed on all study patients. Clinical risk factors and treatment outcomes were assessed.

## Results and discussion

### Results

Over the 15-year period of the study, 68 patients were identified to have both positive corneal and intraocular cultures (anterior chamber and/or vitreous). Of these, six patients were identified to have a culture-proven, delayed-onset endophthalmitis that developed from a culture-positive corneal suture infection.

**Figure 1:**
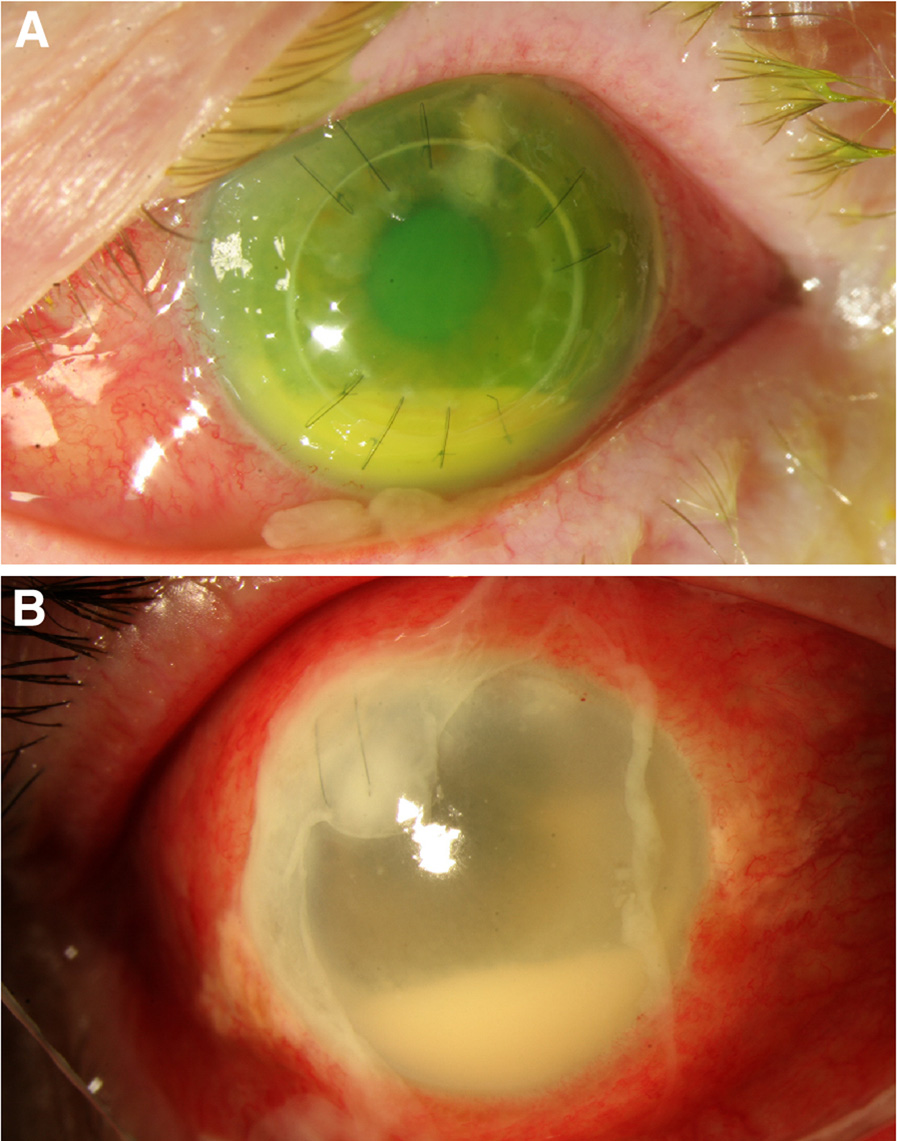
**Representative cases.** (**A**) A 73-year-old woman developed a chronic suture infection and subsequent *Streptococcus salivarius* endophthalmitis after removal of an exposed suture. (**B**) A 74-year-old man developed a suture infection and subsequent *Serratia marcescens* endophthalmitis 2 months following a complicated cataract surgery.

The mean age of patients in the current series was 66.0 years (range, 36 to 74). Three patients were men, and three patients were women. A summary of each patient is provided in Table 1, and representative figures are shown in Figure 1. Five patients had a suture infections related to a previous penetrating keratoplasty wound, and one patient has a suture infection related to a corneal wound from a prior complicated cataract surgery that included a pars plana vitrectomy and intraocular lens placement in the ciliary sulcus. At the time of diagnosis, all patients were using topical prednisolone acetate 1%, with the frequency ranging from hourly to twice daily. Three patients had a history of ocular surface disease. No patients were contact lens users, and no patients had a history of being immunocompromised. Potential contributing mechanisms that were temporally related to endophthalmitis included the removal of a loose or exposed suture (two patients), a broken running suture (one patient), and manipulation of a loose running suture (one patient). In each of these four patients, the corneal infiltrate was present at the time of suture removal or manipulation. One of these patients was not immediately started on topical antibiotics despite the presence of a corneal infiltrate and active manipulation of a suture. After presentation with a corneal infiltrate, topical prednisolone acetate 1% was continued at the same frequency in four patients, decreased in one patient, and stopped altogether in a final patient.

**Table 1:** Demographics and clinical features of patients with corneal suture-related endophthalmitis

**Case number**	**1**	**2**	**3**	**4**	**5**	**6**
Sex/age (years)	Female, 71	Male, 70	Female, 36	Male, 72	Female, 73	Male, 74
Year	1996	1997	1999	2004	2007	2009
Prior surgery	PKP	PKP	PKP	PKP	PKP	CE.IOL
Location of suture abscess	Inferonasal	Superotemporal	Superotemporal	Superotemporal	Superotemporal	Superotemporal
Suture technique	Running	Interrupted	Interrupted	Running	Interrupted	Interrupted
Responsible organism	*Streptococcus pyogenes*	*Streptococcus pneumoniae*	*Streptococcus pneumoniae*	*Streptococcus agalactiae*	*Streptococcus salivarius*	*Serratia marcescens*
Cornea culture result	Positive	Positive	Positive	Positive	Positive	Positive
Suture culture result	Not performed	Not performed	Not performed	Not performed	Positive	Positive
Anterior chamber culture result	Not performed	Not performed	Positive	Positive	Not performed	Not performed
Vitreous culture result	Positive	Positive	Not performed	Not performed	Positive	Positive
Contributing mechanism to suture abscess and endophthalmitis	Loose suture manipulated	Loose suture removed	None identified	Broken running suture	Loose suture removed	None identified
Time from prior surgery to suture abscess diagnosis	59 days	1,324 days	713 days	873 days	365 days	58 days
Time from suture manipulation or complication to endophthalmitis diagnosis	5 days	5 days	NA	4 days	57 days	NA
Topical steroid use at presentation	Prednisolone acetate 1% qid	Prednisolone acetate 1% tid	Prednisolone acetate 1% q1h	Prednisolone acetate 1% qid	Prednisolone acetate 1% bid	Prednisolone acetate 1% qid
Lens status	Pseudophakic	Pseudophakic	Pseudophakic	Pseudophakic	Pseudophakic	Pseudophakic
Intact posterior capsule	Yes	Yes	Yes	No	No	No
Corneal perforation	Yes	No	Yes	Yes	No	No

*PKP* penetrating keratoplasty, *CE.IOL* cataract extraction with intraocular lens implantation, *NA* not applicable, *qid* four times a day, *tid* three times a day, *q1h* every 1 h, *bid* two times a day.

The average number or previous surgeries for all patients was 2.3 (range, 1 to 7). The mean length of time from most recent surgery to presentation with suture infection was 565.3 days (range, 58 to 1,324). In the four patients where there was documented manipulation of a suture or a broken suture, the mean length of time from incident to endophthalmitis diagnosis was 17.8 days (median, 5; range, 4 to 57).

Clinical features at initial diagnosis included corneal infiltrate around an existing suture or previous suture (6/6 [100%]), conjunctival injection (6/6 [100%]), documented vitritis on clinical exam and/or echography (5/6 [83%]), moderate to severe pain (5/6 [83%]), and hypopyon (4/6 [67%]). In the two patients with no documented hypopyon, there was noted to be a full thickness corneal infiltrate with an endothelial plaque and fibrin in the anterior chamber. A corneal perforation or wound dehiscence developed in three of six (50%) study patients.

*Streptoccocus* was identified as the causative organism in five of six patients in the current study. All of the *Streptoccocus* isolates (5/5 [100%]) were sensitive to vancomycin. The single case of *Serratia marcescens* endophthalmitis was sensitive to amikacin, ceftazidime, ciprofloxacin, gentamicin, and tobramycin. No cases of fungal suture infection-related endophthalmitis were identified during the 15-year study period.

Treatment modalities varied and were guided by the attending ophthalmologist depending upon clinical presentation and are shown in Table 2. One patient with severe *Streptococcus pyogenes* keratitis and endophthalmitis underwent a primary enucleation after developing a wound dehiscence. Of the remaining five patients, all received topical and intravitreal antibiotics. Therapeutic penetrating keratoplasty was performed in three patients. Pars plana vitrectomy was performed in two patients. Best corrected visual acuity outcomes at last examination were generally poor and ranged from 20/150 to no light perception.

**Table 2:** Treatment strategies and outcomes in patients with corneal suture-related endophthalmitis

**Case number**	**1**	**2**	**3**	**4**	**5**	**6**
Presenting visual acuity	LP	20/200	LP	LP	20/400	NLP
Management - topical antibiotics	None	Ceftazidime, vancomycin	Gentamicin, vancomycin	Cefazolin, moxifloxacin, vancomycin	Ceftazidime, moxifloxacin, vancomycin	Ceftazidime, vancomycin
Management - intravitreal antibiotics	None	Vancomycin	Vancomycin	Vancomycin	Ceftazidime, vancomycin	Ceftazidime, vancomycin
Management - intravitreal steroids	No	Yes	No	No	Yes	Yes
Management - penetrating keratoplasty	No	No	Yes	Yes	Yes	No
Management - vitrectomy	No	Yes	No	No	Yes	No
Visual acuity at last follow-up visit	NLP/enucleated	LP	LP	20/200	20/150	NLP/enucleated

*LP* light perception, *NLP* no light perception.

## Discussion

Previous studies have detailed potential complications of corneal sutures [1-6]. One large consecutive series by Christo et al. reviewed records of 361 corneal transplant grafts and reported a number of frequent suture-related complications following penetrating keratoplasty including: suture erosions (10.8%), sterile corneal infiltrates (9.4%), and loose sutures in need of surgical repair to prevent wound separation (8.3%) [1]. An ulcerative epithelial defect and infiltrate involving a loose or broken suture developed in 12 grafts (3.3%), but just one of these eyes progressed to endophthalmitis. *Streptococcus* isolates were the most commonly identified organism responsible for suture infections (5/12 patients). Similarly, Leahy et al. reported a series of 18 corneal suture abscesses developing after 773 penetrating keratoplasties (2.3%) [2]. One of these eyes progressed to endophthalmitis after developing a wound separation related to the suture infection. Gram-positive isolates were cultured from 15 of 18 grafts developing a suture infection (83%). *Staphylococcus* species were isolated from ten suture infections, and *Streptococcus* species were isolated from seven suture infections. In 13 of 18 patients, topical steroid use was identified as a possible risk factor for the development of a suture infection.

Another study by Siganos et al. investigated the microbial findings in patients with broken or loose sutures after penetrating keratoplasty [13]. The authors prospectively evaluated 55 eroded sutures in 35 eyes and found that 34 sutures were sterile (61.8%) and 21 sutures had positive cultures for both *Staphylococcus epidermidis* and diptheroids (38.1%). Interestingly, the authors did not report isolating *Streptococcus* species from any of these sutures. An infiltrate was found near 15 of 55 eroded sutures (27.2%) but was less than 1 mm in size in all cases and only seven of the sutures were culture positive. Cultures were not performed on the corneal infiltrate in these cases, and no patients were reported to develop endophthalmitis or severe keratitis resulting in vision loss. Sutures eroded for 24 h or more before removal were statistically more likely to be culture positive in this study (*p* = 0.043).

To date, there have been few patients with corneal suture-related endophthalmitis reported in the literature (Table 3) [7-12]. The series by Christo et al. and Leahy et al. highlighted the rarity of endophthalmitis developing from corneal suture-related infections [1,2]. Additionally, a literature search identified just two previous case series dedicated to the subject of corneal suture infection-related endophthalmitis [7,12]. Khurshid et al. reported a series of five cases of suspected endophthalmitis secondary to suture-related corneal infection [7]. This study was significantly different that the current study in that four patients in their series developed suture infections and endophthalmitis related to extracapsular cataract extraction and one patient developed a suture infection and endophthalmitis related to a corneal laceration repair. All patients in their series failed to improve on topical antimicrobial therapy alone but responded favorably to the administration of intravitreal vancomycin and amikacin. Visual acuity outcomes were very good, with two patients recovering 6/6 vision, one patient recovering 6/7.5 vision, and two patients recovering 6/12 vision. Positive cultures were recovered in two of the five patients, with gram-positive bacteria accounting for both cases (*Streptococcus pneumoniae* and *Staphylococcus aureus*). Additionally, Confino and Brown previously reported a series of three patients who developed bacterial endophthalmitis from a suture abscess [12]. Organisms responsible for endophthalmitis were *Pseudomonas aeruginosa*, *Klebsiella oxytoca*, and *S. epidermidis*. The authors identified loose or degraded sutures in all three cases as the principal source for the development of a suture infection and subsequent endophthalmitis. In this series, visual acuity outcomes were poor and ranged from 20/200 to no light perception.

**Table 3:** Reported cases of bacterial endophthalmitis from corneal suture infections

**First author**	**Confino**	**Leahey**	**Khurshid**	**Current study**
Year of publication	1985	1993	2003	2013
Number of reported cases	3	1	5	6
Number of culture-positive cases	3	1	2	6
PKP-related suture infection	3/3	1/1	0/5	5/6
Cataract wound-related suture infection	0/3	0/1	4/5	1/6
Gram-positive isolates	1/3	1/1	2/2	5/6
Topical steroid use at diagnosis	2/3	1/1	Not reported	6/6
Management - intravitreal antibiotics	1/3	1/1	5/5	5/6
Management - pars plana vitrectomy	1/3	1/1	0/5	2/6
Last visual acuity ≥ 20/50	0/3	0/1	5/5	0/6
Last visual acuity ≥ 20/400	2/3	0/1	5/5	2/6
Enucleation or evisceration	1/3	0/1	0/5	2/6

*PKP* penetrating keratoplasty.

The current study supports the fact that gram-positive bacteria account not only for the majority of cases of corneal suture-related infections but also corneal suture infection-related endophthalmitis. Additionally, while *Staphylococcus* species seem to be more commonly isolated from degraded sutures, it appears that *Streptococcus* species are more often responsible for severe suture infections and endophthalmitis. Furthermore, the current study supports the opinion that the development of endophthalmitis from a suture abscess is uncommon. Despite this fact, potentially modifiable risk factors were identified in all patients who developed endophthalmitis. In four patients, there was documented manipulation of a suture shortly before the development of endophthalmitis, including removal of a loose or exposed suture (two patients), a broken running suture (one patient), or manipulation of a loose running suture (one patient). Additionally, all six of the patients in the current study were using topical steroids, ranging from twice a day to hourly at the time of diagnosis of a suture abscess.

In a 2001 publication, the Royal College of Ophthalmologists recommend that corneal sutures be removed within 3 months following routine extracapsular cataract surgery, even though more recent versions of these guidelines have not provided specific recommendations [14]. Additionally, the decision to remove sutures after a penetrating keratoplasty can be a complex decision, particularly when refractive considerations are involved [15-17]. Regarding suture-related complications following penetrating keratoplasty, Christo et al. advocated the removal of penetrating keratoplasty sutures by 1 year in vascularized recipients and by 18 months in all patients [1]. Other authors have recommended that sutures from corneal grafts preferably be removed within 1 year of the operation or at discharge of patient care [15,16]. In the current study, three patients developed endophthalmitis from a corneal suture infection where the sutures that had been in place for over 2 years following the original surgery. Based on the findings in our study, we hypothesize that the long-term retention of corneal sutures after cataract surgery or penetrating keratoplasty could be a risk factor for endophthalmitis development, although this severe complication is very rare. Furthermore, it appears that prolonged steroid use in cases in which a corneal suture infection is present may increase the risk for progression to endophthalmitis. Alternatives to topical corticosteroids, such as cyclosporin A and/or suture removal should be considered when clinically appropriate. We recommend that topical povidone iodine drops be used prior to suture manipulation in all patients. Additionally, we recommend that patients be placed on topical antibiotic drops after suture manipulation or removal. Patients with infiltrates along corneal sutures should be monitored carefully.

## Conclusions

*Streptococcus* infections accounted for nearly all cases of suture infection-related endophthalmitis in the current study. Topical steroid use and suture manipulation were identified as possible risk factors for developing endophthalmitis. Visual acuity outcomes were poor despite prompt recognition of endophthalmitis and appropriate antibiotic therapy.

## Competing interests

HWF is a consultant for Santen, and ECA is an advisor for Bio-Tissue and receives grant/research support from Alcon, Allergan, and Bausch & Lomb. CRH, DM, ACS, and RKF declare that they have no competing interests.

## Authors’ contributions

CRH participated in study conception, study design, data collection, and drafting of the manuscript. HWF participated in study conception, study design, and drafting of the manuscript. DM is the microbiologist for the study and participated in data collection and critical revision of the manuscript. ACS participated in study conception, data collection, and critical revision of the manuscript. RKF participated in study conception and critical revision of the manuscript. ECA participated in critical revision of the manuscript. All authors read and approved the final manuscript.
